# Plasma-Enabled Selective Synthesis of Biobased Phenolics from Lignin-Derived Feedstock

**DOI:** 10.1021/jacsau.3c00468

**Published:** 2023-11-06

**Authors:** Yichen Ma, Stuart Conroy, Alexander Shaw, Ignacio M. Alliati, Bert F. Sels, Xiaolei Zhang, Xin Tu

**Affiliations:** † Department of Electrical Engineering and Electronics, University of Liverpool, Liverpool L69 3GJ, U.K.; ‡ Department of Chemical and Process Engineering, University of Strathclyde, Glasgow G1 1XJ, U.K.; § School of Mechanical and Aerospace Engineering, Queen’s University Belfast, Belfast BT9 5AG, U.K.; ∥ Center for Sustainable Catalysis and Engineering, KU Leuven, Leuven 3001, Belgium

**Keywords:** Biomass-derived feedstock, Biomass valorization, Nonthermal plasmas, Alkylation, Phenolics

## Abstract

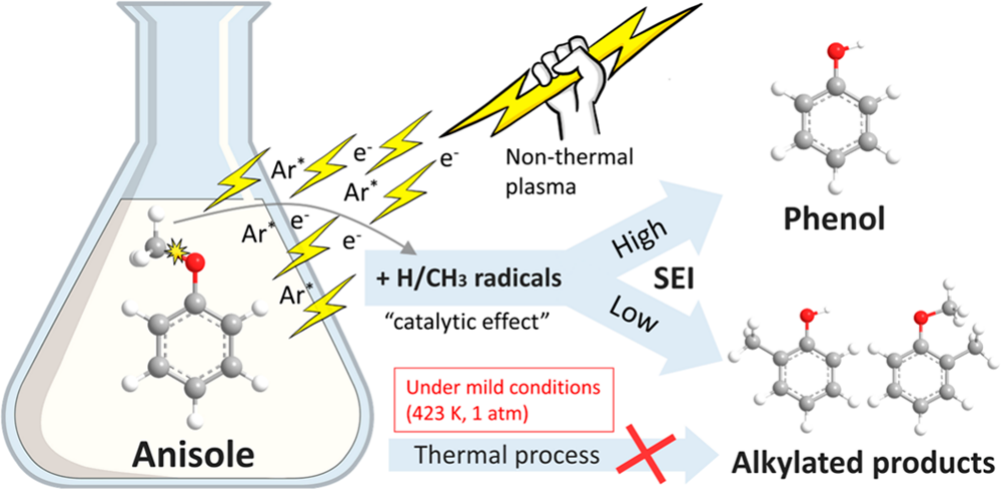

Converting abundant biomass-derived feedstocks into value-added platform chemicals has attracted increasing interest in biorefinery; however, the rigorous operating conditions that are required limit the commercialization of these processes. Nonthermal plasma-based electrification using intermittent renewable energy is an emerging alternative for sustainable next-generation chemical synthesis under mild conditions. Here, we report a hydrogen-free tunable plasma process for the selective conversion of lignin-derived anisole into phenolics with a high selectivity of 86.9% and an anisole conversion of 45.6% at 150 °C. The selectivity to alkylated chemicals can be tuned through control of the plasma alkylation process by changing specific energy input. The combined experimental and computational results reveal that the plasma generated H and CH_3_ radicals exhibit a “catalytic effect” that reduces the activation energy of the transalkylation reactions, enabling the selective anisole conversion at low temperatures. This work opens the way for the sustainable and selective production of phenolic chemicals from biomass-derived feedstocks under mild conditions.

## 1 Introduction

Biomass is an integral part of the global carbon cycle and plays a strategic role in mitigating climate change. Lignin accounts for 25%–35% dry weight of woody biomass and is the only abundant renewable source of aromatics.^1−3^ The valorization of industrial lignin from the waste stream of commercial cellulosic biorefinery, where 60% of lignin is burned as low-value solid fuel, has been well-advocated to improve the biobased economy.^4−7^ Fast pyrolysis is a common approach for lignin utilization, though the lignin-derived bio-oils are compositionally complex, comprising various functional groups, and thus selective defunctionalization strategies are entailed for the synthesis of market-responsive bioproducts. So far, synthetic routes of hydrocarbon fuels from lignin-derived feedstocks have been intensively investigated,^8^ however the scale-up of these routes is limited by excessive H_2_ consumption and low market value of the products compared with other value-added compounds. Thus, new and cost-efficient synthetic streams for valuable bulk or functionalized aromatic chemicals are required to expand the lignin value chain.^9−11^

Phenolics are important platform chemicals in the synthesis of a range of new, drop-in polymer building blocks.^12^ For instance, phenol is one of the critical commodity chemicals that can be used in the packaging and clothing industries.^13^ Phenolics are the building blocks of phenol resins, and phenol is used for the production of bisphenols. As phenolics are currently produced mainly from petroleum-derived or coal-derived feedstocks (e.g., coal tar), the valorization of biomass-derived feedstocks for sustainable synthesis of value-added platform chemicals such as phenolics provides a promising route to support the transition to the zero-carbon circular economy.^14−16^ Nevertheless, most of the existing synthetic routes require rigorous reaction conditions (i.e., high temperature and/or high pressure) and high-cost hydrogen (Table S1), while still facing significant challenges including waste disposal, corrosion, and catalyst deactivation by coking and sintering.^17−19^ Therefore, developing innovative and sustainable technologies for the selective synthesis of phenolic bioproducts from lignin-derived feedstocks under mild conditions has attracted increasing attention.

Nonthermal plasma (NTP) technology provides an emerging and promising alternative to traditional catalytic processes for the transformation of lignin-derived feedstocks into chemicals under mild conditions. During NTP processes, the bulk gas kinetic temperature remains low, while highly energetic electrons with a mean electron energy of 1–10 eV are initially generated, which enables the activation of reactants (e.g., anisole) and background gas to form a cascade of chemically reactive species such as excited atoms, ions, and molecules that could facilitate chemical reactions.^20−22^ This unique nonequilibrium feature of NTP enables thermodynamic unfavorable chemical reactions to proceed at atmospheric pressure and low temperatures,^23−27^ thus, to avoid using high temperature and/or high pressure required in catalytic or thermal processes. In addition, plasma processes can be switched on and off instantly, offering the flexibility to be combined with intermittent renewable energy sources (e.g., wind and solar energy) for decentralised plasma electrification toward chemical energy storage.^28^

Despite these favorable prospects, NTP has been so far used in the activation of lignocellulose but limited in the conversion of lignin-derived feedstocks,^29−31^ and it remains a significant challenge due to the complex chemistry involved in this process. Anisole was chosen as a model compound for its simplicity and relevance to lignin-derived compounds, specifically those containing methoxy moieties. While anisole may not encompass the entire complexity of lignin-derived mixtures, it serves as a useful and well-defined model compound for studying plasma-assisted conversion of lignin-derived compounds. To date, the reaction mechanism in the plasma transformation of biomass-derived feedstocks is unknown, while the role of plasma generated reactive species in these processes is not clear, both of which limit the potential for the tunable and selective synthesis of targeted chemicals from biomass-derived feedstocks. An atomic level understanding of this synthesis process using a combination of *in situ* plasma diagnostics, density functional theory (DFT) calculations and plasma kinetic modeling would offer a promising way to elucidate the reaction pathways of hydrogen-free anisole-to-phenol-and-cresol conversion and to get new insights into the potential role of plasma-induced reactions in the selective and tunable synthesis of chemicals from biomass-derived feedstocks.

Here, we report a highly selective, tunable, and hydrogen-free plasma process for the synthesis of phenolics from anisole at ambient pressure and low temperature (∼150 °C). The influence of key processing parameters, anisole feed rate, Ar flow rate, discharge power, and specific energy input (SEI) on the plasma synthesis process was evaluated to explore the feasibility for tuning the selectivity of phenolic bioproducts. A unique combination of *in situ* spectroscopic diagnostics, DFT calculation, and plasma kinetic modeling was developed to gain new insights into different reaction pathways for the selective plasma synthesis of phenolics from lignin-derived anisole.

## 2 Results and Discussion

### 2.1 Plasma-Enhanced Synthesis of Phenolics

**Figure 1:**
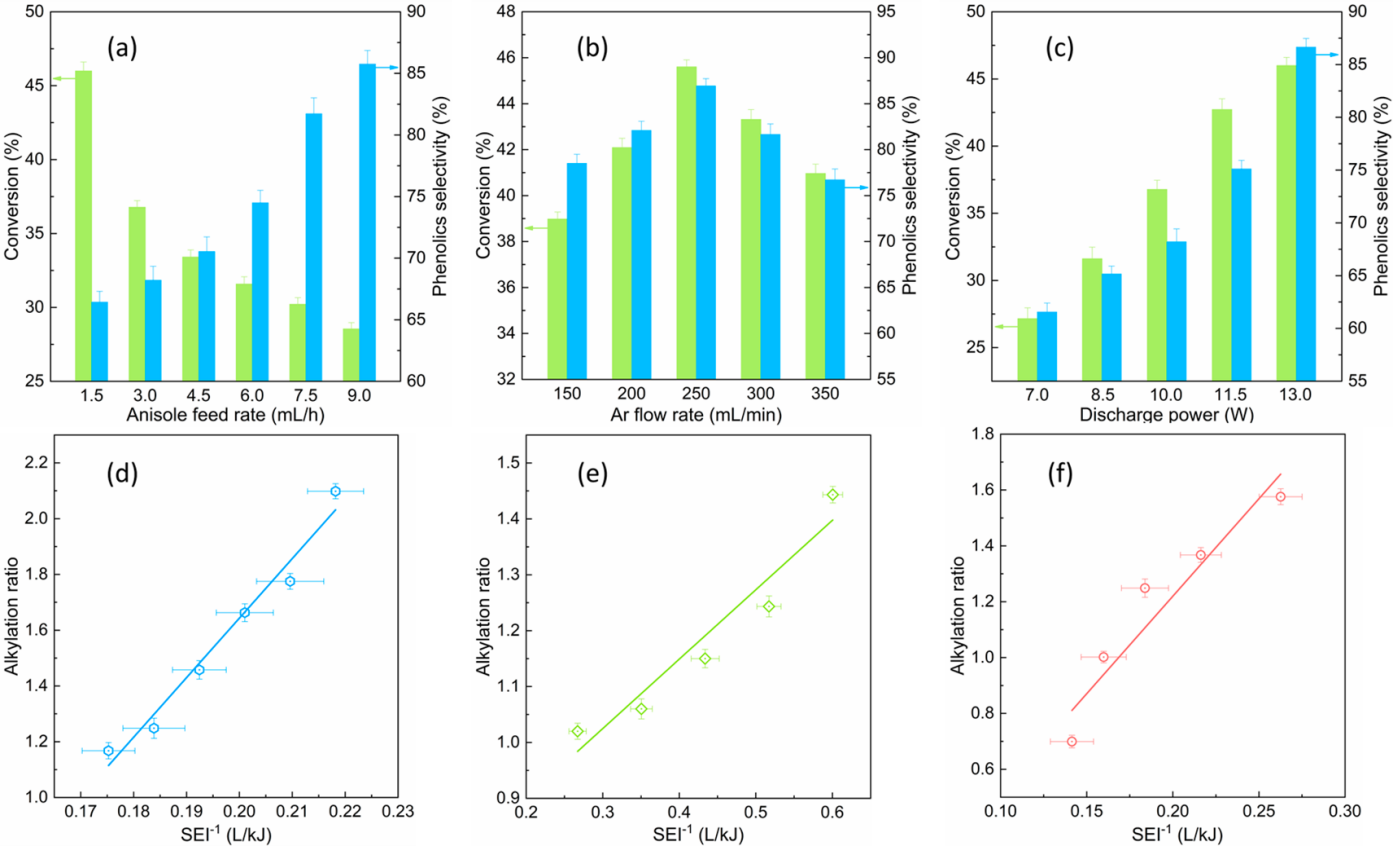
Performance of plasma-enhanced conversion of anisole. Influence of (a) anisole feed rate, (b) Ar flow rate, and (c) discharge power on the conversion of anisole and selectivity of phenolics. (d)–(f) Alkylation ratios as a function of reciprocal SEI for different process parameters in (a)–(c).

Figure 1 shows the effect of anisole feed rate, Ar flow rate, and discharge power on the anisole conversion and phenolics selectivity. Phenolic compounds (phenol and cresols) and methylanisoles were found as the dominant products (Figures S1–S3 and Table S2), while BTX (benzene, toluene, and xylenes) was also produced with low selectivity. The CO_*x*_-free methane-rich gas (mainly CH_4_ and H_2_) was formed with a total selectivity of 2.0–3.0%; thus, gas production in this process is insignificant and will not be the focus in this work.

Increasing the anisole feed rate from 1.5 to 9.0 mL/h enhances the selectivity of phenolics from 66.4% to 85.8% (Figure 1a), while the selectivity of phenol and cresols varies from 41.4% to 29.3% and 25.1% to 56.5%, respectively (Figure S1b). The conversion of anisole decreases with the anisole feed rate, which can be attributed to the decreased plasma power density per anisole molecule at a fixed discharge power. In contrast, the energy efficiency for the conversion of anisole increases almost linearly with an increase in anisole feed rate (Figure S1a). A clear trade-off between energy efficiency and anisole conversion can be found. High anisole feed rate contributes to low energy cost due to the increased converted anisole despite the decrease of anisole conversion.

Figure 1b shows that the conversion of anisole increases with the elevated Ar flow rate and reaches a plateau at 250 mL/min, while further increasing the flow rate (to 350 mL/min) substantially reduces the performance of the plasma process. The optimum flow rate is found as 250 mL/min to maximize the conversion of anisole (45.6%), selectivity of phenolics (86.9%), and energy efficiency (136.1 g/kWh) simultaneously. As shown in Table S1, this plasma process can achieve high phenolics selectivity at low temperature without using high-cost hydrogen in comparison to thermal catalysis processes. Generally, a higher argon flow rate lowers the anisole concentration and thus enhances the energy dissipated on each reactant molecule. However, increasing the argon flow rate also decreases the SEI and the residence time of anisole in the plasma zone, reducing the collisions of anisole with energetic electrons and reactive species. Therefore, the effect of argon flow rate on the conversion of anisole and energy efficiency is strongly dependent on the balance between these opposite effects: (i) enhanced anisole conversion due to the positive effect of lowered anisole concentration; and (ii) reduced anisole conversion due to the negative effect induced by the decreased SEI and residence time.

Figure 1c shows that the conversion of anisole is nearly doubled from 27.2% to 46.0% when increasing the discharge power from 7 to 13 W. The selectivity of phenolics shows a similar evolution to the conversion of anisole, rising from 61.6% to 86.7%, while the phenol selectivity increases from 34.9% to 53.2% (see Figure S3b). Increasing power dissipated in the plasma area at a fixed residence time enhances the total number of filaments produced in the discharge. The increased formation of microdischarges can create more reaction channels and reactive species, facilitating the plasma conversion of anisole.^32^ Nevertheless, the energy efficiency shows an opposite trend, decreasing moderately with the discharge power. Thus, balancing anisole conversion and energy efficiency is important for the further technological development of this process.

Retaining the methyl groups in the anisole transformation can effectively improve the atom economy. The alkylation ratio is determined based on the abundance of alkylated products to evaluate the alkylation-related reactions in the plasma process. When increasing the anisole feed rate to 6.0 mL/h, cresols replace phenol as the dominant products (see Figure S1), suggesting the synthesis of alkylated compounds can be tuned by changing the anisole feed rate. As shown in Figures 1e and S2, increasing the argon flow rate from 150 to 350 mL/min significantly reduces the SEI from 3.7 to 1.7 kJ/L but simultaneously increases the alkylation ratio from 1.0 to 1.5. Conversely, increasing the discharge power simply lowers the alkylation ratio. These findings indicate that the formation of alkylated compounds is suppressed at a low anisole feed rate, low argon flow rate, and high discharge power; these all have one common feature, high specific energy input. Interestingly, we found that the alkylation ratio is independent of anisole conversion or product selectivity but closely related to the SEI.

As shown in Figure 1d–f, a strong negative correlation can be identified between the alkylation ratio and the SEI. A similar finding was reported in thermal catalytic conversion of 4-ethylphenol that isomerization and transalkylation are thermodynamically favorable at low temperatures.^33^ In this study, our results reveal the relationship between SEI and alkylation-related reactions, providing a route for tuning the selectivity to alkylated products by SEI for future investigations of the plasma process.

### 2.2 Role of Plasma-Generated Species

#### 2.2.1 Electron Properties

**Figure 2:**
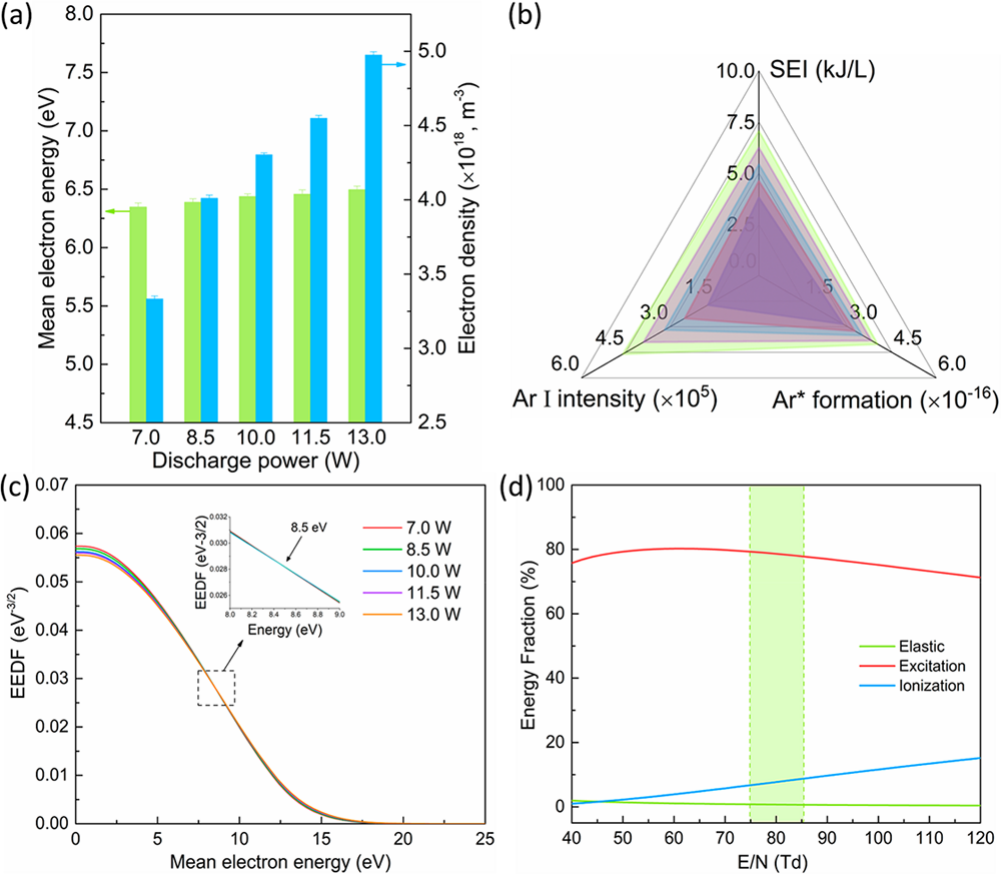
Relationships between the energy density and plasma-induced electrons and reactive species. (a) Calculated mean electron energy and electron density as a function of discharge power. (b) Scheme of the ternary relationship among the SEI (corresponding to the discharge power), relative intensity of Ar atomic line at 763.51 nm from OES, and calculated rate constants of Ar* formation reactions. (c) Calculated EEDF under different reduced electric fields E/N. (d) Energy fraction consumed in different electron-impact reactions of Ar as a function of the reduced electric field E/N (green zone illustrates the range of operating conditions in this study). NTP system: Ar flow rate 100 mL/min; anisole feed rate 3.0 mL/h.

The conversion of anisole is initiated by plasma-induced highly energetic electrons. Insights into the properties of electrons (i.e., electron temperature and density) could shed light on the reaction pathways. The methods for the calculation of electron properties are given in Supporting Information Section 4. As shown in Figure 2a, increasing discharge power enhances the electron density while the mean electron energy remains constant at around 6.4 eV. The maximum electron density reaches 5.0 × 10^18^ m^–3^ at a discharge power of 13.0 W. In Figure 2c, the electron energy distribution functions (EEDFs) prove that most electrons possess electron temperatures within the range of 0–10 eV, and the intersection of the curves takes place at around 8.5 eV. According to the EEDFs and improved electron density, increasing the discharge power generates more high-energy electrons. To summarize, enhancing SEI produces more highly energetic electrons that enhances the formation of more chemically reactive species involving in the activation of anisole.

#### 2.2.2 Ar Excited Species

Ground-state Ar species can be activated to the metastable states through collision with highly energetic electrons (R1). As seen in Figure 2d, the energy fraction consumed in the Ar excitation reactions dominates among different electron-impact reactions within the range of the operating conditions. Previous studies emphasized the significance of metastable states of working gas to the plasma chemical processes,^34^ and more specifically, the metastable argon can effectively enhance the conversion of reactants by creating otherwise infeasible reaction pathways.^35^ Accordingly, we believe both the electrons and electron induced metastable Ar contribute to the anisole conversion; even so, the electron energies are more likely to be consumed in the formation of argon excited species.
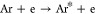
R1The formation of Ar excited species can be confirmed by the strong Ar atomic lines in the emission spectra (Figure S4). The relative intensity of the Ar atomic line at 763.51 nm was used as a probe to evaluate the prevalence of Ar excited species.^36^Figure 2b demonstrates the ternary relationship among the SEI, the relative intensity of the Ar atomic line at 763.51 nm, and the rate coefficients for Ar^*^ generation reactions. Notably, the rate coefficients of Ar^*^ formation and the population of Ar excited species correspond well with the SEI. As the rates of the electron impact reactions are strongly correlated with the abundance of electrons, increasing SEI improves the electron density and therefore facilitates the transformation of argon into other internal degrees of freedom.^37^

These findings suggest that the excited Ar atoms are the key reactive species in plasma, and the abundance of these species is closely related to the SEI. Increasing SEI enhances the electron density and the prevalence of Ar metastable states, which facilitates the cleavage of etheric C–O bonds in anisole and thus reduces the formation of alkylated products in this process.

#### 2.2.3 Vibrational Excited Anisole Molecules

**Figure 3:**
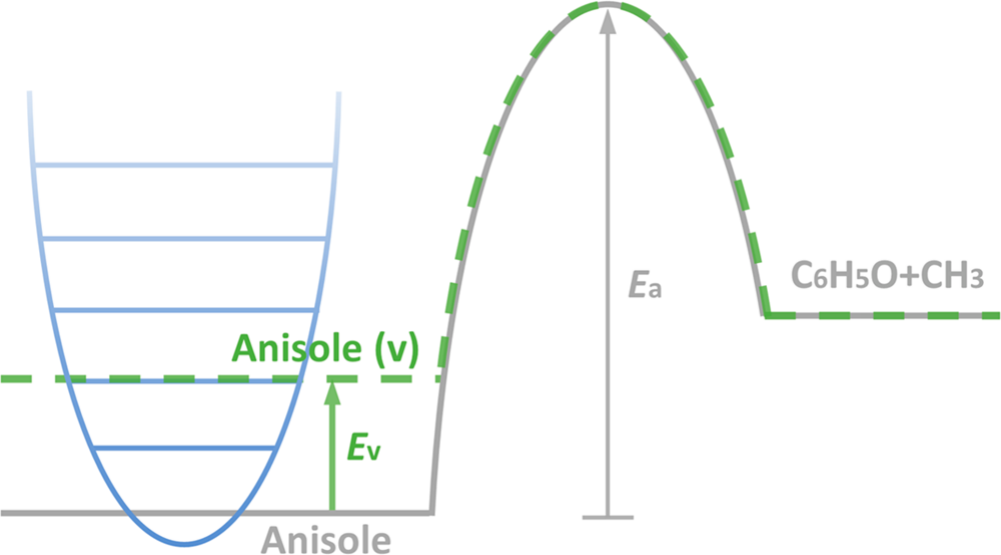
Schematic reaction coordinate for the dissociation of anisole from ground state (gray line) and vibrationally excited state (green dashed line).

Besides the argon atoms, the reactant molecules can also be activated by chemically reactive species in the plasma. More specifically, the dominant metastable argon may activate anisole molecules prior to their transformation mainly via the vibrational excitation, as the molecular vibrational excitation significant contributes to plasma chemical conversion.^38^ Energy from electron impact may also be deposited into vibrational modes that are orthogonal to the relevant reaction coordinate. Therefore, the activation of anisole could contribute to an increased initial energy and the capability to traverse an otherwise inaccessible pathway. This enhancement to the reaction can be orders of magnitude higher than thermal reactions under mild conditions.^39^Figure 3 illustrates the reaction coordinates for a typical anisole dissociation incorporating this effect. The initial energy of a ground state anisole (gray curve) can be effectively enhanced via vibrational excitation (green dashed curve) and therefore lowers the energy barrier of anisole dissociation. This reduction of activation energy may contribute significantly to the reaction rate of anisole conversion.

To further explore this concept, we developed a numerical method to incorporate plasma-induced vibrational excitations of anisole. The enhancement factor *F* was introduced to investigate the effect of molecular vibrations on the rate constants (detailed calculation shown in Supporting Information Section 6). Table 1 demonstrates a set of enhancement factors, *F* of anisole dissociation reactions calculated from different vibrational modes. By incorporating this effect, the initial energies of anisole up to 37.1 kJ/mol can be achieved from the ground state to the first vibrationally excited state (vibrational quantum number *v* = 0 to *v* = 1), indicating the rates of anisole dissociation would be considerably elevated in conjunction with vibrational excitation. In this respect, the rate constants of C–H bond dissociation at the methyl group and the benzene ring can be magnified by up to 4 orders of magnitude. At other positions of anisole, the probabilities of dissociation are also multiplied to some degree. Note that the molecular vibration is of minimal significance to the C-OCH_3_ bond, revealing the weak impact of the plasma-generated reactive species on the cleavage of this bond. In the context of its intrinsic property (high BDE shown in Table 2), the combined effect of bond strength and weak vibration contributes to the low selectivity to arenes and considerable production of phenolics in this plasma process.

**Table 1:** Enhancement Factor *F* for Different Vibrational Modes of Anisole at a Gas Temperature of 423 K

Vibrational mode	*F*
CH_3_ stretch	2.7 × 10^4^
O–CH_3_ stretch	3.4 × 10^1^
C-OCH_3_ torsion	1.3 × 10^0^
aromatic CH stretch	3.8 × 10^4^
aromatic CC stretch	2.2 × 10^2^

**Table 2:** Comparison of Homolytic BDEs in Anisole Molecules

Group	BDE (kJ/mol)
PhO–Me	290.2
Ph–OMe	426.9
PhOCH_2_–H	404.0
H–PhOMe	473.6, 464.0, 469.0

### 2.3 Reaction Pathways of Plasma-Enhanced Anisole Conversion

#### 2.3.1 Bond Dissociation Energies

It is worth noting that phenolics are the dominant products in the plasma process. Understanding the inherent properties of anisole can extend fundamental insights into this phenomenon. From the thermal pyrolysis standpoint, the chemical bond dissociations are typically endothermic, where the energy barriers are closely related to the thermal stabilities of reactant molecules. The thermal stabilities of bonds in an anisole molecule can be identified by their bond dissociation energies (BDEs) which are useful probes to indicate the strength of a chemical bond. The distinction of BDEs determines the probabilities of single bond fissions, leading to different reaction rates at each location of the reactant molecules.

The investigation of BDEs in the reactant molecule can help reveal the reaction mechanism from the energetics of different types of chemical bonds. As the inherent aromatic properties of molecules are preserved in this work, we mainly concentrate on the BDEs of the C–O and C–H bonds in anisole. Table 2 shows a comparison of the homolytic BDEs in anisole computed at 298.15 K using the M06-2X functional with the Def2-TZVP basis set. The low BDE of the PhO–Me bond (290.2 kJ/mol), i.e., high reactivity of the etheric C–O bond in anisole, validates the high selectivity of phenolics in this work (Ph: phenyl group). In comparison with the methyl group (404.0 kJ/mol), the hydrogen abstraction from the benzene ring (464.0–473.6 kJ/mol) is found more strenuous, indicating H radicals in the system are more likely sourced from the methyl group. To summarize, the order of BDEs (H–PhOMe > Ph–OMe > PhOCH_2_–H > PhO–Me) in anisole reveals that the cleavage of the methyl group is prone to occur, which corresponds well with the distribution of liquid products. It is noteworthy that the above-mentioned findings that aryl C–O bonds (e.g., Ph–OH and Ph–OMe) are stronger than etheric C–O bonds (e.g., PhO–Me) were proved true regardless of the number or type of additional substituent groups.^40,41^

#### 2.3.2 Proposed Reaction Pathways

**Figure 4:**
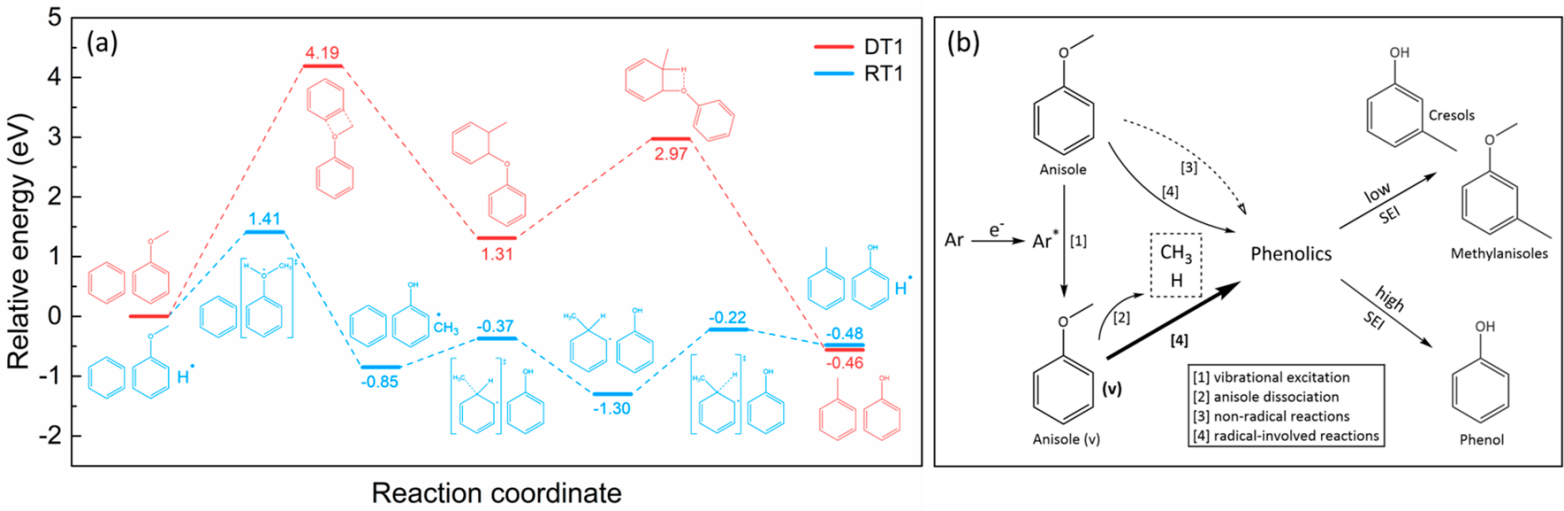
(a) Energy profiles for the direct transalkylation reaction DT1 and radical transalkylation reaction RT1. (b) Proposed reaction pathways of the plasma-enhanced anisole conversion in Ar plasma (solid lines denote probable pathways, while dashed line means less probable route; the thickness of lines is given based on reaction rates).

To gain insights into the reaction mechanism of this plasma transformation process, DFT calculations were performed. The Gibbs free energies of activation and reaction rate constants were calculated for possible pathways of the anisole conversion in terms of anisole dissociation, direct transalkylation (DT), radical transalkylation (RT), radical substitution (RS), and hydrogen atom transfer (HT) (see Supporting Information Sections 7–10). In general, the conversion of anisole originates from anisole dissociation reactions facilitated by plasma-induced vibrational excitations. Given the abundant aromatic products in this study, the rupture of the benzene ring is considered less probable. Instead, unimolecular decompositions of anisole (R2–R7) that preserve the inherent aromatic properties are proposed as the initial reactions in the plasma. These reactions are likely to be the major source of CH_3_/H radicals participating in the subsequent radical-induced reactions (Table S3).
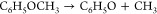
R2
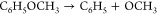
R3
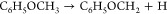
R4
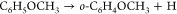
R5
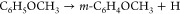
R6
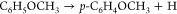
R7In addition, the importance of bimolecular transalkylation reactions have also been frequently emphasized in the anisole conversion.^42−45^ To gain better insights into the underlying mechanisms, we investigated four possible direct transalkylation pathways, DT1-DT4, as shown in R8–R11, respectively. Each pathway involves two elementary reactions, both proceeding through a 4-centered transition state (see Supporting Information Section 8). However, the rate coefficients of these reactions were found to be insignificant at a low temperature of 423 K (Table S4), further emphasizing the unimolecular decomposition reactions (R2–R7) dominate the initial anisole conversion in this plasma process (An: anisole, Ben: benzene, Tol: toluene, Ph: phenol, Cr: cresols, MA: methylanisoles),
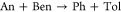
R8
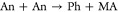
R9
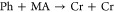
R10
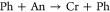
R11Compared with the traditional thermal or catalytic process, the presence of abundant highly reactive species in NTP could not only increase the initial energy of the reactant but also create new reaction routes. More specifically, in the presence of plasma-induced hydrogen or methyl radicals, the DT reactions can be considerably facilitated by being transferred into the RT pathways. A comparison of energy profiles for DT1 pathway and RT1 pathway is given to elaborate on this mechanism, as shown in Figure 4a. The DT1 route is initiated by the cleavage of methoxy O–CH_3_ bond in anisole, forming a transition state bonding with benzene at methyl and oxygen-bearing fragments (Figure S5). The second step involves the rupture of the newly formed C–O bond to generate phenol and toluene. The whole pathway of DT1 has an activation energy of 4.19 eV. In the RT1 pathway, the first elementary reaction involves the hydrogen radical attack on the methoxy oxygen to form phenol and a methyl radical, followed by a two-step radical substitution to yield toluene and a hydrogen radical. Notably, the corresponding activation barrier of RT1 is substantially lower (1.41 eV), indicating a more kinetically favorable reaction pathway. To further confirm this concept, the general mechanism of other possible DT and RT reactions are illustrated in Figures S6–S8.

Interestingly, a “catalytic effect” of the plasma-generated radicals can be clearly identified from the radical transalkylation reactions. For instance, in pathway RT1 the hydrogen radical undergoes initial sequestration and subsequent regeneration. Reordering of the pathway such that the toluene formation precedes the anisole conversion leads to an equally valid reaction mechanism, and in that instance, the methyl radicals would act as a “catalyst”. This “catalytic effect” can also be observed in RT2-RT4 where each pathway is catalyzed by either a hydrogen atom or a methyl radical. It is considered as the decisive factor for considerably enhanced rate coefficients of the radical-induced transalkylation reactions compared with direct transalkylation. Furthermore, this effect relating to the plasma-induced radicals marks an exceptional property of this plasma process that enables the low-temperature transformation of anisole that would not be possible otherwise.

In addition to transalkylation reactions, a broad involvement of the radicals can be observed throughout the entire process. Originally, the radicals are mainly generated in the anisole dissociation reactions before participating in radical substitution, hydrogen transfer, and other general reactions. The detailed description of these reactions in terms of the general schemes for pathways, free energies of activation, and rate constants are elaborated in the Supporting Information Sections 9 and 10 (Tables S5 and S6; Figures S9–S13). In our model, the radical transalkylation can be regarded as the integration of several elementary reactions. To simplify the calculation in the chemical kinetic modeling, the rate constants of radical transalkylation were summarized from radical substitution reactions using steady-state approximations and equilibrium state approximations.

#### 2.3.3 Chemical Kinetics

To further explore the underlying mechanisms of the entire reaction network, a kinetic analysis was performed using the 0D ZDPlasKin model incorporating the above-mentioned possible pathways and vibrational excitations of anisole. Embedding the enhancement factors of plasma-induced vibrational excitations of anisole (Table 1) into the rate constants of anisole conversion reactions, the conversion of anisole could be significantly enhanced. Figure S14a shows the number density of major species derived from the plasma chemical kinetic modeling. The increasing number density signifies that the whole system is far from equilibrium after 100 ms. This time slot was then chosen to qualitatively investigate the mechanisms prior to equilibrium. The calculated anisole conversion and product selectivity are plotted in Figure S14b where the phenolics remain the main product, corresponding with the experimental data.

In Figure S14c, the major processes of anisole loss are evaluated by integrating the amount of converted anisole molecules through different reactions to elucidate the role of major pathways, especially of the transalkylation pathways. It can be easily observed that the radical transalkylation reactions are magnitudes faster than the anisole dissociation and the direct transalkylation. Although significant in the traditional anisole transformation, the direct transalkylation reactions almost stagnate at a low bulk temperature. Meanwhile, the plasma-induced radicals can effectively accelerate the conversion of anisole with their unique “catalytic effect” in radical transalkylation reactions. The reactions also account for a large proportion of phenolic synthesis routes in this work. Some other reactions, hydrogen transfer, for instance, may reach a considerable forward reaction rate but with very limited contribution to the anisole conversion due to its rapid reverse reaction. The incorporation of plasma kinetics with the DFT results leads us to conclude that the radical transalkylation is the dominant pathway involved in this plasma-enhanced anisole conversion process.

Based on these discussions, plausible reaction pathways of plasma-enhanced anisole conversion are proposed in Figure 4b. Through drastic collision with plasma-induced highly energetic electrons, ground-state Ar species are activated to the metastable states. The anisole molecules can be vibrationally excited by the electrons and metastable argon. This vibrational excitation weakens the energy barrier of anisole dissociation reactions where the hydrogen and methyl radicals are mainly generated. These small radicals exhibit favorable “catalytic effects” that significantly facilitate the transalkylation reactions. The radical transalkylation reactions are therefore found as the dominant pathway in the plasma anisole conversion. In addition, broad involvement of the plasma-generated radicals can be observed in the radical-induced reactions, while the nonradical reactions also take place. The product distribution can be tuned by varying the specific energy input. High SEI could promote the electron temperature and the abundance Ar metastable states, which facilitate the dissociation of PhO-Me bonds in anisole and therefore improve the phenol formation. At a low SEI, the majority of the methyl groups are retained, and the conversion of anisole to cresols can be achieved with 100% atom economy.

## 3 Conclusion

In summary, we have demonstrated a noncatalytic and hydrogen-free plasma process that enables the selective conversion of biomass-derived anisole into value-added phenolics bioproducts at low temperature (∼150 °C) and ambient pressure. The highest phenolic selectivity of 86.9% and the energy efficiency of 136.1 g/kWh were achieved at an anisole conversion of 45.6%. More interestingly, the alkylation ratio was found independent of anisole conversion or product selectivity but negatively correlated with SEI, which enables the tuning of selectivity of the alkylated compounds by SEI. The electrical and *in situ* emission spectroscopic diagnostics reveal that increasing SEI produces more highly energetic electrons that enhances the formation of Ar metastable species, both of which contribute to the enhanced activation of anisole, especially the vibrational excitations. Small reactive species such as hydrogen and methyl radicals exhibit a favorable “catalytic effect” to facilitate the transalkylation reactions. The plasma kinetic modeling shows the radical-induced reactions are the dominant pathway in the anisole conversion. Lignin transformation involves a diverse array of compounds, and our findings provide valuable insights into the plasma-assisted conversion of lignin derivatives and open a new route for the sustainable and selective synthesis of higher value platform chemicals from biomass-derived anisole. This study can serve as a foundation for further research to explore a broader range of more complex lignin-derived compounds to investigate the selective breaking of C–C and C–O.

## 4 Methods

### 4.1 Experimental Setup

**Figure 5:**
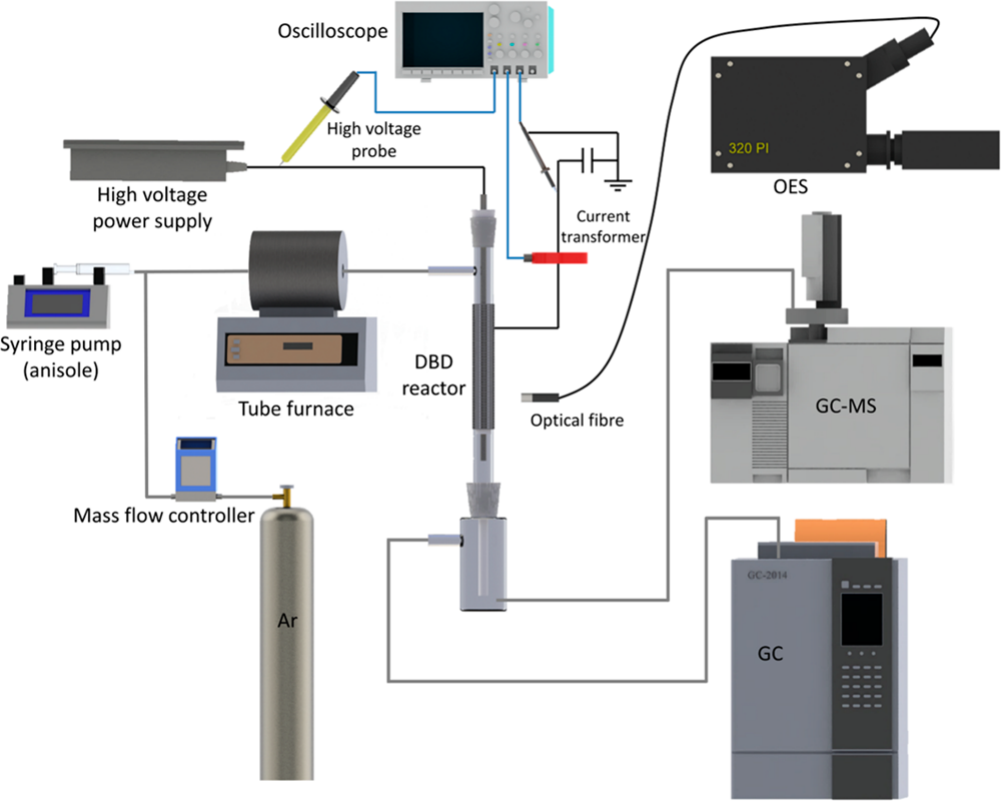
Schematic diagram of the experimental setup.

Figure 5 shows a schematic diagram of the experimental setup. A typical coaxial DBD reactor was developed for the conversion of biomass-derived anisole. A quartz tube with an inner diameter of 10 mm and a wall thickness of 2 mm was used as the dielectric layer. A stainless-steel mesh (outer electrode) of 50 mm length was wrapped around the quartz tube, while a stainless-steel rod with an outer diameter of 4 mm served as a concentric inner high voltage electrode. The DBD reactor was connected to a high voltage AC power supply with a frequency of 9.2 kHz and a maximum peak voltage of 30 kV. The applied voltage was measured by a high voltage probe (Testec, TT-HVP 15 HF), while the current was recorded with a current monitor (Magnelab CT-E0.5). Both signals were sampled by a four-channel digital oscilloscope (Tektronix, DPO2024B). The discharge power was calculated by using the Lissajous figure obtained from the oscilloscope. Pure argon (99.999%, BOC) was used as the carrier gas and controlled with a mass flow controller (Omega, FMA-2404). Anisole (99%, ACROS Organics) was injected into a tube furnace using a high-precision syringe pump (KDS Legato, 100) (Carbolite, MTF 12/38/250 1200 °C), and was preheated to 373 K and mixed with argon before passing into the reactor. The temperature of the typical working condition (∼150 °C) was measured by a fiber optical thermometer (Omega, FOB102) placed into the discharge area.

### 4.2 Product Analysis and Data Evaluation

The effluent lines of reactor were terminated with a liquid trap containing 10 mL of acetone (99.8%, Fisher Chemical) to dissolve the liquid products. After each experiment, the reactor was rinsed with another 5 mL of acetone. The collected samples were quantified by gas chromatography–mass spectrometry (Agilent 7820A MSD 5975C) and identified by using a mass spectral library from the National Institutes for Standards and Technology (NIST). Gas products were measured by gas chromatography (Shimadzu GC-2014) equipped with dual detectors. The emission spectra in the range 200–900 nm were recorded by using a spectrometer with an intensified CCD (ICCD) camera (Princeton Instruments, 320 PI). Each experiment was repeated 3 times. Generally, the margin of error in this work was within 3%.

The conversion of anisole (*X*) was defined as
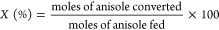
1As all the quantified liquid products in this work are aromatic compounds, the selectivity of product *i* can be calculated as
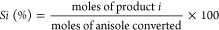
2To evaluate the reactions toward alkylated aromatics, the alkylation ratio was defined in eq 3.
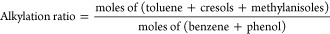
3The specific energy input (SEI) was determined in eq 4.
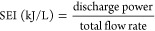
4The energy efficiency (η_e_) of the anisole conversion can be calculated as
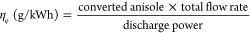
5

### 4.3 Computational Methods

To gain insight into the reaction mechanism, we performed density functional theory (DFT) calculations of the primary reaction pathways and the bond-dissociation energies (BDEs) at different positions of the lignin-derived model compound. All calculations were performed using Gaussian 16 (Rev A.03),^46^ employing the M062X functional^47^ with the Def2-TZVP basis set.^48^ The unrestricted formalism was used for the calculation of all open-shell species. The dispersion was included in the form of Grimme’s D3 empirical dispersion correction^49^ without any damping scheme. Frequencies calculations were performed at a temperature of 298.15 K for BDE calculations and 423.15 K for rate constant calculations. Standard pressure was used throughout. Transition state structures were verified through observation of a single negative frequency mode and, where necessary for clarification, intrinsic reaction coordinate calculations.

Bond dissociation enthalpies were calculated using the following equation:
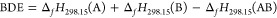
6where A and B represent the radicals formed from the homolytic dissociation of the molecule AB. Δ_*f*_*H*_298.15_ is the enthalpy of formation of the species under standard conditions.

The rate constants (*k*) were calculated using the Eyring equation in the form:
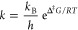
7where *k*_B_ is Boltzmann’s constant, *h* is Planck’s constant, Δ^‡^*G* is the Gibbs free energy of activation, *R* is the gas constant, and *T* is the temperature. The value of Δ^‡^*G* is determined as the difference in free energies between the transition state and ground-state.

Based on the DFT under transition state theory, a 0D plasma kinetic model was developed to elucidate the underlying mechanisms. The ZDPlasKin Fortran module with an integrated BOLSIG+ solver was employed to calculate the rate constants of the plasma reactions.^50^ This chemical kinetic model involves 264 reactions and 70 species. The reaction rate coefficients of the primary reactions were determined by DFT calculations. In addition to these reactions, we compiled a few reactions from conventional chemical kinetic models^51,52^ to perform a convincing simulation for the entire reaction network. A coaxial DBD zero-dimensional model^32^ was employed in conformity with the experimental conditions in this study.

## Supporting Information Available

Comparison of plasma and thermal catalysis in synthesis of biobased phenolics, energy efficiency and product distributions, identified liquid products from GC-MS, plasma calculation by means of BOLSIG+, spectroscopic characteristics, enhancement of anisole dissociation through vibrational excitation, anisole dissociation reactions, transalkylation pathways, radical substitution reactions, hydrogen atom transfer reactions, kinetic simulations (PDF)
