# Supplementary material for: Plasma-Enabled Selective Synthesis of Biobased Phenolics from Lignin-Derived Feedstock
Source: JACS Au. 2023 Nov 6;3(11):3101–10. doi: 10.1021/jacsau.3c00468 (PMC10685411; doi:10.1021/jacsau.3c00468)
Supplement: Supplementary file 1 — au3c00468_si_001.pdf [file au3c00468_si_001.pdf]

Supporting Information for

Multiperspective Decoupling Analyses between Global Embodied Carbon Chains and Global Value Chains

Yuhan Liang [1], Qiumeng Zhong [1], Zijun Deng [1], Hui Li [2], Jetashree [2], Zhifeng Yang [1], Sai Liang [1,*]

1 Key Laboratory for City Cluster Environmental Safety and Green Development of the Ministry of Education, School of Ecology, Environment and Resources, Guangdong University of Technology, Guangzhou, Guangdong, 510006, China

2 School of Environment, Beijing Normal University, Beijing 100875, China

* Correspondence to: liangsai@gdut.edu.cn (Sai Liang).

Number of Pages: 8

Number of Figures: 3

The Supporting Information provides additional information on: (1) decoupling degree; (2) sensitivity analysis; and (3) supplementary results.

Figure S1 Decoupling statuses categorized by decoupling metric

Figure S2 Comparisons of the decoupling results calculated by consumption-based GHG emissions and consumption-based value added and that calculated by consumption-based GHG emissions and GDP during 1995-2019

Figure S3 Comprehensive indexes of carbon inequality of nations in the global trade network from the consumption and income perspectives

S1 Decoupling degree

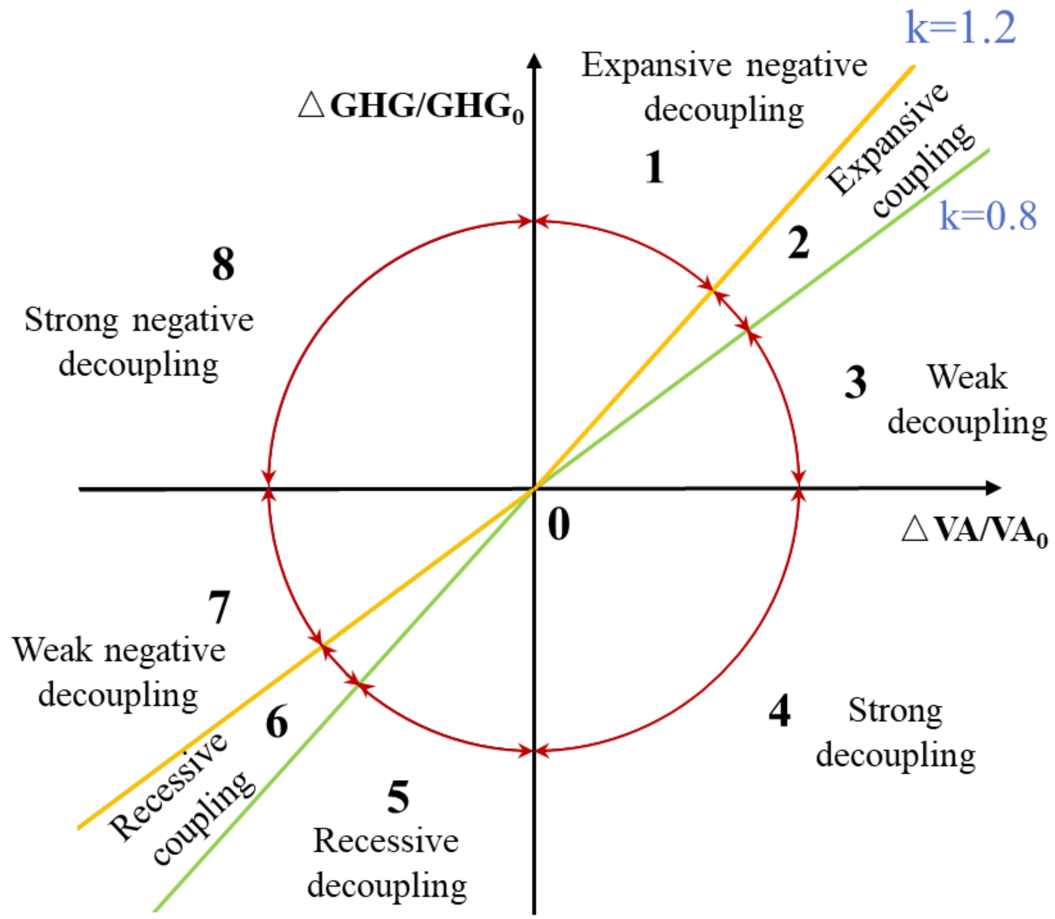

Figure S1. Decoupling statuses categorized by decoupling metric.

The decoupling statuses can be categorized into eight degrees and three types:

1) negative decoupling: ① expansive negative decoupling (△GHG%>0, △V%>0, DI>1.2), ⑦ weak negative decoupling (△GHG%<0, △V%<0, 0<DI<0.8), and ⑧ strong negative decoupling (△GHG%>0, △V%<0, DI<0);

2) coupling: ② expansive coupling (△GHG%>0, △V%>0, 1.2>DI>0.8) and ⑥ recessive coupling (△GHG%<0, △V%<0, 1.2>DI>0.8);

3) decoupling: ③ weak decoupling (△GHG%>0, △V%>0, 0<DI<0.8), ④

strong decoupling ($\triangle$GHG%<0, $\triangle$V%>0, DI<0), and ⑤ recessive decoupling ($\triangle$GHG%<0, $\triangle$V%<0, DI>1.2).

The decoupling statuses are stronger than the coupling and negative decoupling. The strong decoupling status (i.e., GHG emissions decreased along with the economic growth) is desired by sustainable development. The expansive negative decoupling, expansive coupling, and weak decoupling statuses indicate that GHG emissions increased along with economic growth and the difference lies in their increasing rates. The recessive decoupling, recessive coupling, and weak negative decoupling indicate that GHG emissions decreased with the economic downturn. The strong negative decoupling indicates that GHG emissions decreased along with the economic downturn, which is undesired by sustainable development.

S2 Sensitivity analysis

We evaluated the sensitivity of GHG emissions induced by the final demand to all the parameters (e.g., GHG emission intensity, intermediate transaction matrix, and final demand) by calculating their sensitivity coefficients and elasticities. The elasticities equal to the ratios between the changing rates of GHG emissions and the changing rates of the parameters. They are used to evaluate how these three variables affect the GHG emissions. For example, the notation $\boldsymbol{GHG_c}$ indicates national GHG emissions induced by the final demand; vector $\boldsymbol{f_G}$ denotes the GHG emission intensity of province sectors; $\mathbf{L}$ indicates the Leontief inverse matrix; and vector $\boldsymbol{y_i}$ means the final demand of province i. Scalars $\frac{\partial GHG_c}{\partial f_{Gi}}$ and $\frac{\partial GHG_c}{\partial y}$ indicate the sensitivity coefficients for GHG emission intensity and final demand; and $\frac{\partial GHG_c}{\partial \mathrm{T}_{ij}}$ indicates the sensitivity coefficients of intermediate transaction matrix. Scalars $\frac{\frac{\partial GHG_c}{GHG_c}}{\frac{\partial f_{Gi}}{f_{Gi}}}$ and $\frac{\frac{\partial GHG_c}{GHG_c}}{\frac{\partial y_j}{y_j}}$ indicate the elasticities for GHG emission intensity and final demand; and $\frac{\frac{\partial GHG_c}{GHG_c}}{\frac{\partial \mathrm{T}_{ij}}{\mathrm{T}_{ij}}}$ indicate the elasticities for intermediate transaction matrix.

$$\boldsymbol{GHG_c} = \boldsymbol{f_G} \times (\mathbf{I} - \mathbf{A})^{-1} \times \boldsymbol{y_i} = \boldsymbol{f_G} \times \mathbf{L} \times \boldsymbol{y_i} \tag{S1}$$

$$\frac{\partial GHG_c}{\partial f_{Gi}} = (\mathbf{L} \times \boldsymbol{y})_i \tag{S2}$$

$$\frac{\frac{\partial GHG_c}{GHG_c}}{\frac{\partial f_{Gi}}{f_{Gi}}} = (\mathbf{L} \times \boldsymbol{y})_i \times \frac{f'_{Gi}}{GHG_c} \tag{S3}$$

$$\frac{\partial GHG_c}{\partial \mathrm{T}_{ij}} = \frac{(f_G \times \mathrm{L})_i \times (\mathrm{L} \times y)_j}{X_j} \tag{S4}$$

$$\frac{\frac{\partial GHG_c}{GHG_c}}{\frac{\partial \mathrm{T}_{ij}}{\mathrm{T}_{ij}}} = \frac{(\boldsymbol{f}_G \times \mathbf{L})_i \times (\mathbf{L} \times \boldsymbol{y})_j}{X_j} \times \frac{\mathrm{T}_{ij}}{GHG_c} \tag{S5}$$

$$\frac{\partial GHG_c}{\partial \boldsymbol{y}} = \boldsymbol{f}_G \times \mathbf{L} \tag{S6}$$

$$\frac{\frac{\partial GHG_c}{GHG_c}}{\frac{\partial y_j}{y_j}} = (\boldsymbol{f}_G \times \mathbf{L})_j \times \frac{y_j}{GHG_c} \tag{S7}$$

Results show that the parameter with the highest sensitivity is the final demand of construction in China (0.11). It means that the induced GHG emissions would change by 1.1% if the final construction demand in China changed by 10%. Other parameters with relatively high sensitivity include the GHG emission intensity of the *electricity by coal* (0.06) and *basic iron and steel and of ferro-alloys and first products thereof* sectors (0.03) in China.

S3 Supplementary results

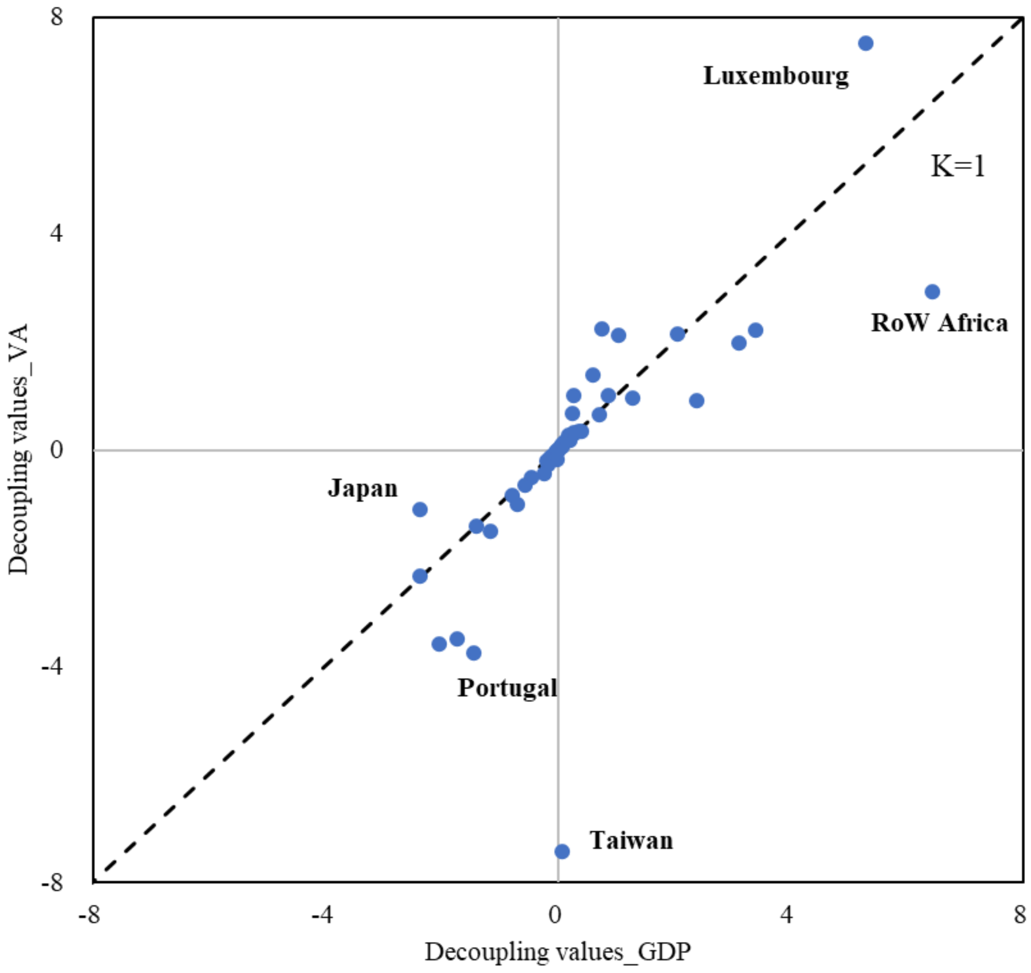

Figure S2. Comparisons of the decoupling results calculated by consumption-based GHG emissions and consumption-based value added and that calculated by consumption-based GHG emissions and GDP. The horizontal axis indicates the decoupling values calculated by consumption-based GHG emissions and GDP. The vertical axis indicates the decoupling values calculated by consumption-based GHG emissions and embodied value added.

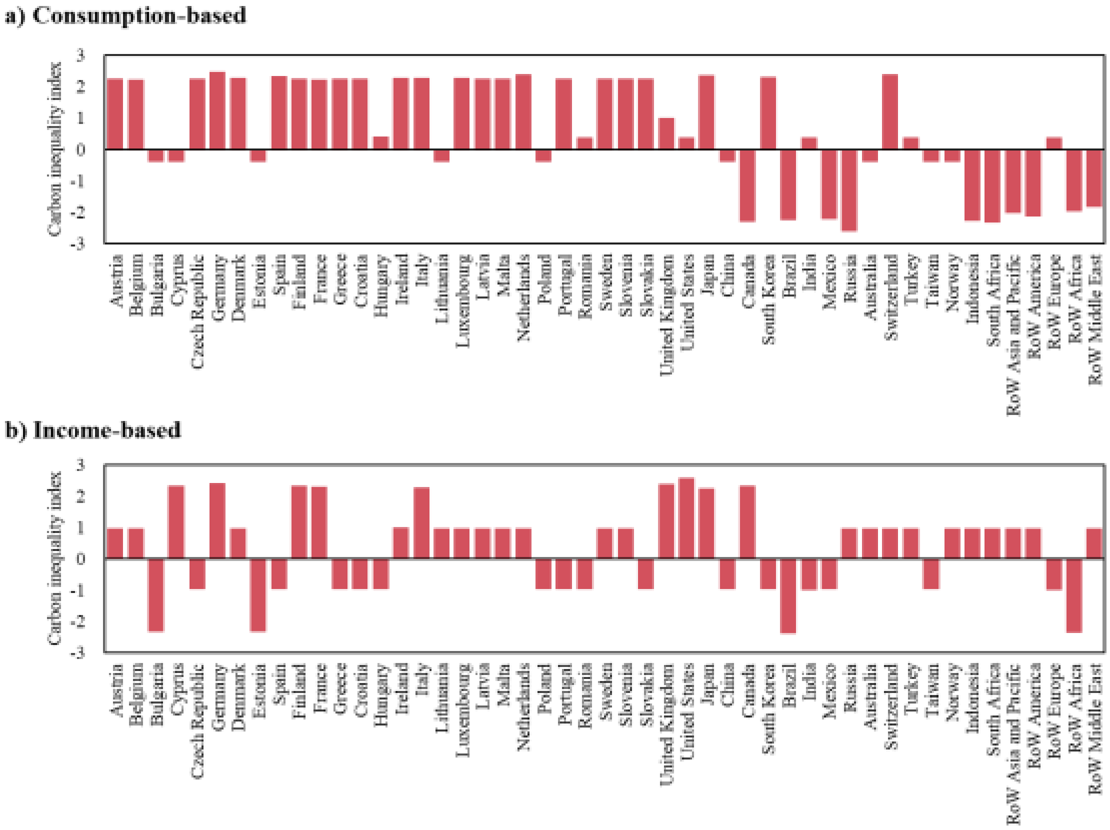

Figure S3. Comprehensive indexes of carbon inequality of nations in the global trade network from the consumption and income perspectives.
